# End-to-end robot intelligent obstacle avoidance method based on deep reinforcement learning with spatiotemporal transformer architecture

**DOI:** 10.3389/fnbot.2025.1646336

**Published:** 2025-10-08

**Authors:** Yuwen Zhou, Weizhong Zhang

**Affiliations:** ^1^School of Engineering Mathematics and Technology, University of Bristol, Bristol, United Kingdom; ^2^Anhui Xinhua University, Hefei, China

**Keywords:** deep reinforcement learning, spatiotemporal attention mechanism, transformer architecture, end-to-end obstacle avoidance, autonomous robot navigation

## Abstract

To enhance the obstacle avoidance performance and autonomous decision-making capabilities of robots in complex dynamic environments, this paper proposes an end-to-end intelligent obstacle avoidance method that integrates deep reinforcement learning, spatiotemporal attention mechanisms, and a Transformer-based architecture. Current mainstream robot obstacle avoidance methods often rely on system architectures with separated perception and decision-making modules, which suffer from issues such as fragmented feature transmission, insufficient environmental modeling, and weak policy generalization. To address these problems, this paper adopts Deep Q-Network (DQN) as the core of reinforcement learning, guiding the robot to autonomously learn optimal obstacle avoidance strategies through interaction with the environment, effectively handling continuous decision-making problems in dynamic and uncertain scenarios. To overcome the limitations of traditional perception mechanisms in modeling the temporal evolution of obstacles, a spatiotemporal attention mechanism is introduced, jointly modeling spatial positional relationships and historical motion trajectories to enhance the model's perception of critical obstacle areas and potential collision risks. Furthermore, an end-to-end Transformer-based perception-decision architecture is designed, utilizing multi-head self-attention to perform high-dimensional feature modeling on multi-modal input information (such as LiDAR and depth images), and generating action policies through a decoding module. This completely eliminates the need for manual feature engineering and intermediate state modeling, constructing an integrated learning process of perception and decision-making. Experiments conducted in several typical obstacle avoidance simulation environments demonstrate that the proposed method outperforms existing mainstream deep reinforcement learning approaches in terms of obstacle avoidance success rate, path optimization, and policy convergence speed. It exhibits good stability and generalization capabilities, showing broad application prospects for deployment in real-world complex environments.

## 1 Introduction

With continuous breakthroughs in artificial intelligence (Chen et al., 2025), automatic control, and environmental perception technologies (Ni et al., 2025), the autonomous navigation and obstacle avoidance capabilities of robots have become key factors in enabling intelligent mobile robotic systems (Ullah et al., 2024). Especially in various typical application scenarios such as warehouse logistics, urban delivery, agricultural operations, and public services, robots are often required to operate continuously in dynamic, complex, and even unknown environments, placing higher demands on their path planning and obstacle avoidance decision-making capabilities (Katona et al., 2024). Meanwhile, the uncertainty, diversity, and real-time nature of these environments pose significant challenges to the perception, reaction speed, and robustness of robotic systems (Wang et al., 2021).

Against this background, intelligent obstacle avoidance technologies for robot groups have rapidly developed and become a hot research topic in the robotics field. Specifically, path planning technologies are responsible for identifying a feasible route from a starting point to a target point within a given map or environment, while obstacle avoidance technologies require robots to dynamically perceive and avoid both static and dynamic obstacles during movement, achieving local or global path adjustment and optimization (Almazrouei et al., 2023). Traditional methods mostly rely on a sequential “mapping-then-planning” framework, which, while effective in structured environments, often faces issues such as high computational complexity, delayed responses, and strong dependence on environmental stability when dealing with scenarios featuring randomly appearing obstacles or frequent environmental changes (Guo et al., 2022).

In complex scenarios, robots must simultaneously handle various types of static and dynamic obstacles, such as stacked objects, pedestrians, other mobile devices, and obstacles resulting from unexpected events (Zheng et al., 2022). Traditional path planning methods often struggle to cope with such situations. For instance, heuristic algorithms like the widely used A algorithm (Liu et al., 2022) demonstrate strong feasibility and controllability in static environments but suffer from inefficiencies and poor adaptability in dynamic or time-sensitive applications. Additionally, intelligent optimization methods such as Ant Colony Optimization (ACO; Miao et al., 2021) and Particle Swarm Optimization (PSO; Shami et al., 2022) have improved path quality and global search capability to some extent, but still face bottlenecks such as unsmooth paths, susceptibility to local optima, and slow convergence rates, making them inadequate for modern robots operating in highly dynamic environments.

On the other hand, traditional obstacle avoidance frameworks typically adopt modular processing pipelines, where perception, mapping, path planning, and control are handled in separate stages (Qin et al., 2023). While this design offers clear structure and relatively low engineering implementation costs, in practical deployments, accumulated errors between modules, delays in information transmission, and a lack of global modeling capability often degrade obstacle avoidance performance. Particularly in unstructured or semi-structured environments such as farmlands, mountainous areas, narrow corridors, or temporary warehouse setups, it is difficult to completely model feasible paths in advance, limiting the adaptability of traditional methods to diverse and uncertain scenarios. As application scenarios become increasingly complex and expectations for robot intelligence continue to rise, building intelligent obstacle avoidance systems with enhanced environmental awareness, dynamic adaptability, path quality assurance, and decision-making optimization has become a key research direction for mobile robotics (Lahn et al., 2023).

In recent years, Deep Reinforcement Learning (DRL) has emerged as a prominent approach in robot obstacle avoidance research due to its strong autonomous learning capabilities and adaptability (Dai et al., 2024). Especially DRL models based on algorithms like Deep Q-Network (DQN) can continuously interact with the environment to learn optimal obstacle avoidance strategies in complex scenarios, eliminating the need for prior modeling and handcrafted rules (Issa et al., 2021). However, when handling high-dimensional sensory data and dynamic obstacle distributions, relying solely on perception modules based on traditional convolutional neural networks often fails to capture temporal dependencies and global environmental features, limiting the generalization and robustness of learned policies.

To address these challenges, this paper proposes an end-to-end intelligent robot obstacle avoidance method that integrates deep reinforcement learning with a spatiotemporal Transformer architecture. This method adopts DQN as the core for policy learning, introduces the powerful global modeling capability of the self-attention mechanism in Transformers, and incorporates a spatiotemporal attention structure to jointly perceive and model the spatial distribution and temporal evolution of obstacles, thereby improving the adaptability of decision-making strategies to dynamic environmental changes (Phatak et al., 2023). The proposed architecture establishes an integrated end-to-end learning pipeline from perception to decision-making, effectively overcoming the limitations of traditional methods in multi-stage processing and local feature modeling.

Before detailing our contributions, we justify our choice of DQN over other DRL frameworks. While PPO and A3C offer superior sample efficiency and stability in continuous action spaces, and SAC excels in stochastic environments, DQN provides several advantages for our specific application: (1) discrete action spaces are more suitable for robot navigation commands (move forward, turn left/right, stop), (2) the Q-value function naturally aligns with our spatiotemporal feature representation from the Transformer, (3) the experience replay mechanism enables efficient utilization of our diverse simulation data, and (4) the deterministic policy output ensures consistent obstacle avoidance behaviors essential for safety-critical applications.

The contributions of this paper are as follows:

1. Deep Q-Network (DQN) is introduced as the core framework for robot obstacle avoidance strategy learning, constructing an adaptive learning path from perceived states to action decisions. Traditional obstacle avoidance methods rely on handcrafted rules or supervised signals and struggle with continuous decision-making in dynamic, complex, or unknown environments. In contrast, by combining DQN with environmental state learning to approximate Q-value functions, robots can autonomously optimize obstacle avoidance strategies through interaction with the environment without requiring explicit models. Compared with static strategies or conventional Q-learning, DQN utilizes deep neural networks to achieve high-dimensional state space mapping, significantly improving the generalization and decision-making efficiency of obstacle avoidance behaviors. Furthermore, experience replay and target network update mechanisms stabilize the learning process and prevent policy oscillations.
2. A spatiotemporal attention mechanism is incorporated as a crucial component for perception modeling and policy optimization in obstacle avoidance tasks, aiming to simultaneously capture key spatial features and temporal evolution patterns in the environment. Traditional perception modules tend to focus only on local spatial information at the current moment, lacking dynamic modeling capabilities for historical states, resulting in instability when dealing with moving obstacles or complex scenarios. To address this, this paper proposes a fused attention structure combining temporal and spatial information. By embedding a spatiotemporal joint attention module during feature encoding, the model can dynamically focus on critical obstacles, path boundaries, or motion trends across different time points and spatial regions. This mechanism constructs dependencies between local and global information, enabling the model to more accurately identify potential collision risks and safe passages without losing important contextual information.
3. An end-to-end Transformer architecture is designed as the core modeling framework for the robot obstacle avoidance system, aiming to overcome the information fragmentation and feature loss issues caused by traditional separated perception-decision structures. Compared with previous approaches relying on handcrafted feature extraction or stacked convolutional-recurrent networks, the proposed Transformer-based framework offers powerful global modeling capabilities and parallel processing efficiency. It can capture long-range dependencies of critical obstacles and potential paths in multi-scale spatial environments. Specifically, the Transformer encoder performs sequential modeling of spatiotemporal information, while the decoder directly outputs action policies or value functions, providing high-quality semantic representations for deep reinforcement learning. This Transformer-based architecture not only improves the generalization and response speed of obstacle avoidance strategies in dynamic and complex scenarios but also significantly reduces reliance on intermediate modules or manually designed features, demonstrating excellent end-to-end intelligent obstacle avoidance performance.

The structure of this paper is organized as follows:

Section 2 reviews related work and discusses prior research, summarizing their strengths and limitations. Section 3 details the proposed method, including the DQN framework, spatiotemporal attention mechanism, and Transformer architecture, along with explanations of the algorithmic workflow. Section 4 presents the experimental setup, comparative evaluations, ablation studies, and visualized results. Finally, Section 5 discusses the conclusions, limitations of this study, and outlines directions for future research.

## 2 Related work

We organize our analysis of existing obstacle avoidance methods along four critical dimensions: (1) perception mechanism and feature extraction capabilities, (2) reinforcement learning framework and policy optimization strategy, (3) architectural integration level and information flow design, and (4) temporal modeling and sequential dependency handling. This systematic framework allows us to identify specific gaps that our proposed method addresses.

In dynamic, variable, and unstructured environments, the primary challenges faced by robots in obstacle avoidance include perception uncertainty, the complexity of environment modeling, the real-time nature of path planning, and the robustness of decision-making behavior (Wijayathunga et al., 2023). Traditional methods, such as path planning algorithms based on A, Dijkstra, and Ant Colony Optimization, have demonstrated certain effectiveness in structured environments. However, these approaches typically require static modeling of the environment and rely on accurate maps, lacking the adaptability to handle unexpected obstacles and dynamic changes. Moreover, these algorithms commonly suffer from limitations such as tortuous paths, slow convergence, and sensitivity to local optima, making them inadequate for complex, real-world scenarios.

To address the limitations of traditional methods, the research community has introduced Simultaneous Localization and Mapping (SLAM) techniques (Alsadik and Karam, 2021), enabling robots to achieve self-localization and environment modeling in unknown environments. Significant progress has been made in SLAM, from classical Extended Kalman Filter (EKF)-based methods to enhanced algorithms like Particle Filter (PF) and Rao-Blackwellized Particle Filter (RBPF; Zhang, 2022). The Gmapping algorithm proposed by (Grisetti et al. 2007), a representative of RBPF-SLAM, achieved high-precision 2D map construction. With advances in visual perception technologies, Visual SLAM (VSLAM) has emerged as a research hotspot, integrating robust image feature detection algorithms such as SIFT and SURF, and has been widely applied in object recognition and 3D mapping (Ansari, 2019; Ni et al., 2024). However, SLAM still faces challenges in practical applications, including slow processing speeds and sensitivity to feature occlusion and lighting variations, which limit its obstacle avoidance responsiveness in highly dynamic environments.

In recent years, the rise of deep learning has brought new paradigms to robotic obstacle avoidance. As research in this field has progressed, increasing efforts have been dedicated to enhancing the real-time performance, accuracy, and generalization capability of obstacle avoidance systems to meet the demands of complex and variable environments (Raghvendra et al., 2024). Notably, the emergence of transformer-based reinforcement learning approaches, including Decision Transformers (Chen et al., 2021) and Trajectory Transformers (Janner et al., 2021), has introduced sequence modeling paradigms to RL that show promise for handling temporal dependencies in robotic decision-making. These attention-based architectures have demonstrated effectiveness in offline RL settings and long-horizon planning tasks, though their application to real-time obstacle avoidance remains limited due to computational constraints and the need for specialized spatial reasoning mechanisms. From the evolution of recent work, it is evident that research paradigms have gradually expanded from rule-driven approaches to data-driven, structure-integrated, and task-specialized directions, with transformer-based methods representing the latest frontier in end-to-end learning approaches.

Firstly, explorations into lightweight visual perception are of significant practical value. For instance, the method proposed in (Misir and Celik 2025), based on an improved MobileNetV2 for visual obstacle avoidance, designed a neural network with low parameter volume, combined with a custom dataset integrating color intensity and ultrasonic data, achieving high recognition accuracy and successful deployment on a mobile robot platform (Zhang et al., 2022). This demonstrates considerable practicality and engineering feasibility. However, the closed nature of its experimental scenes and data construction limits the model's generalization ability in complex and dynamic environments. Furthermore, it lacks quantitative analysis regarding algorithmic latency and computational load, posing reliability challenges for real-world applications. Meanwhile, task-specialized pathways for robotic obstacle avoidance have also demonstrated notable advantages. For example, (Jian et al. 2025) focused on surgical robotics and proposed the STV-PDNN structure, integrating velocity control optimization and null-space redundancy control, achieving high-precision and stable trajectory control during surgical procedures with excellent policy robustness and trajectory continuity. However, its validation scope is narrow, confined to specific procedures and structures, lacking comprehensive evaluations in diversified anatomical scenarios and effective strategies for handling dynamic obstacles and sensor interference. From the perspective of control and trajectory optimization integration, (Li et al. 2025b) and (Wang et al. 2025b) proposed trajectory tracking methods combining Model Predictive Control (MPC) with Sliding Mode Control, and an integrated planning-tracking obstacle avoidance framework, respectively. Both approaches improved system performance through soft and hard constraint modeling, optimized planning, and controller design. Notably, (Wang et al. 2025b) enhanced system adaptability in unstructured environments by introducing dynamic obstacle intention modeling into optimization objectives, breaking the conventional hierarchical design. Nevertheless, both studies suffer from limited application scenarios, simplified dynamic obstacle modeling, and incomplete complexity analysis, which restrict their scalability to real-world multi-target, multi-obstacle interactive environments. In the field of coordinated obstacle avoidance for multiple manipulators in open environments, (Wu et al. 2025) proposed a dual-arm collaborative obstacle avoidance method, incorporating obstacle classification mechanisms and avoidance direction adjustment strategies. It exhibited strong flexibility and collision avoidance capabilities during practical task execution, especially for fine-grained obstacle avoidance control in complex operation scenarios. However, this method has yet to adequately address the complexity and dynamic diversity of obstacles in extreme environments. It also lacks in-depth investigation into system robustness under sensor errors and has not conducted quantitative comparisons with mainstream methods, affecting the comprehensiveness of its practical value verification.

To systematically position our contribution, we identify four key limitations in existing approaches: (1) Perception Limitations: Most methods rely on CNN-based local feature extraction or simple attention mechanisms that fail to capture long-range spatiotemporal dependencies crucial for dynamic obstacle prediction, (2) Integration Gaps: Traditional approaches maintain separation between perception and decision modules, leading to information loss and suboptimal policies, (3) Temporal Modeling Deficiencies: Existing methods either ignore temporal dynamics entirely or use limited memory mechanisms (LSTM) that suffer from gradient vanishing in long sequences, and (4) Scalability Issues: Many approaches are tested only in simplified scenarios and lack comprehensive evaluation across diverse environments. Our method addresses these limitations through: unified spatiotemporal modeling via Transformer architecture, complete end-to-end integration eliminating information bottlenecks, comprehensive temporal dependency modeling without gradient issues, and extensive validation across multiple challenging environments.

Based on our systematic analysis, while existing works demonstrate progress in individual components, they exhibit fundamental limitations in achieving true end-to-end spatiotemporal modeling for dynamic obstacle avoidance in the practical deployment of obstacle avoidance algorithms, control precision, and task adaptability, revealing a multi-level integration trend from visual perception and motion control to policy optimization. However, several unresolved issues remain: most methods have been validated in limited environments, lacking systematic evaluations for dynamic, unstructured, and multi-obstacle interactive scenarios; obstacle avoidance systems often decouple perception and decision-making, leading to response delays and increased system complexity; and some lightweight or end-to-end solutions lack coordinated optimization of hardware resources and real-time performance. Against this backdrop, this paper aims to develop an end-to-end deep reinforcement learning obstacle avoidance framework integrating a spatiotemporal attention mechanism. Leveraging the advantages of the Transformer architecture in sequential modeling and global perception, combined with the adaptive learning capability of policy optimization, this study addresses the decision-making challenges of obstacle avoidance in densely dynamic environments, filling the current research gap between sequential modeling capacity, dynamic adaptability, and deployment-friendliness.

Although considerable achievements have been made in SLAM and DRL, VSLAM and image processing, as well as path planning and control strategies, there is still a lack of a method capable of integrating environment perception, spatiotemporal sequence modeling, and autonomous policy learning into a unified, end-to-end obstacle avoidance framework without relying on explicit mapping. Existing methods often adopt decoupled designs for perception, mapping, and control, resulting in complex systems, difficult deployments, and delayed responses. To address this research gap, this paper proposes an end-to-end robot intelligent obstacle avoidance method based on deep reinforcement learning and a spatiotemporal Transformer architecture. This approach employs Transformer as the backbone model, incorporating spatial attention mechanisms to enhance environmental obstacle modeling and integrating DRL for self-optimized policy learning, aiming to achieve an efficient, robust, and generalizable robotic obstacle avoidance system in dynamic environments.

## 3 Method

**Figure 1:**
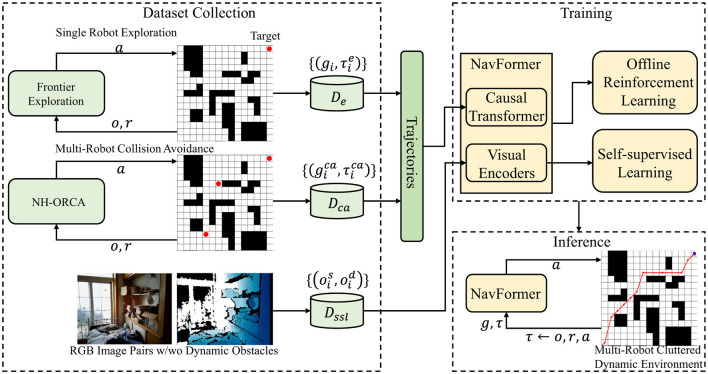
Overall algorithm architecture. NH-ORCA (Non-Holonomic Optimal Reciprocal Collision Avoidance) generates training data for multi-robot collision avoidance scenarios.

In the data collection phase, NH-ORCA (Non-Holonomic Optimal Reciprocal Collision Avoidance) algorithm is employed for multi-robot collision avoidance to generate training trajectories that account for the kinematic constraints of wheeled mobile robots. The overall architecture is shown in Figure 1.

Figure 1 illustrates the complete workflow of our proposed end-to-end obstacle avoidance system, which consists of three interconnected phases: dataset collection, training, and inference. In the dataset collection phase (left panel), we generate diverse training scenarios through two pathways: single robot exploration using frontier exploration techniques to create basic navigation data {*g*_*i*_, τ_*i*_}, and multi-robot collision avoidance scenarios using NH-ORCA (Non-Holonomic Optimal Reciprocal Collision Avoidance) algorithm to produce complex interaction data $\left\{g_{i}^{\text{ca}},\tau_{i}^{\text{ca}}\right\}$. Additionally, we collect RGB image pairs with and without dynamic obstacles $\left\{o_{i}^{s},o_{i}^{d}\right\}$ to form our comprehensive dataset $D_{\text{ssl}}$. The training phase (top right) integrates our NavFormer architecture with both offline reinforcement learning and self-supervised learning mechanisms, where the Causal Transformer processes the collected trajectories and visual encoders handle the multimodal sensory inputs. The inference phase (bottom right) deploys the trained NavFormer model in real-time, taking current observations (*o, r*) and generating appropriate actions (*a*) for multi-robot cluttered dynamic environments. The bidirectional arrows indicate the iterative feedback between training and data collection, enabling continuous improvement of the obstacle avoidance policy.

### 3.1 Deep Q-network

The end-to-end architecture integrates data collection, feature extraction, and decision-making in a unified learning framework, eliminating the need for separate perception and planning modules typical in conventional robotic systems.

Our implementation incorporates several key extensions to the standard DQN framework to adapt it to spatio-temporal obstacle avoidance tasks. When integrated with Transformer-based feature extraction, the Q network processes high-dimensional spatio-temporal features rather than raw observation data. The modified experience replay algorithm maintains the sequence dependencies required for dynamic obstacle tracking by preserving temporal consistency. Therefore, by considering obstacle distance-adaptive greedy exploration for safer exploration and multi-scale reward shaping, we simultaneously balance immediate collision avoidance and long-term navigation efficiency.

**Figure 2:**
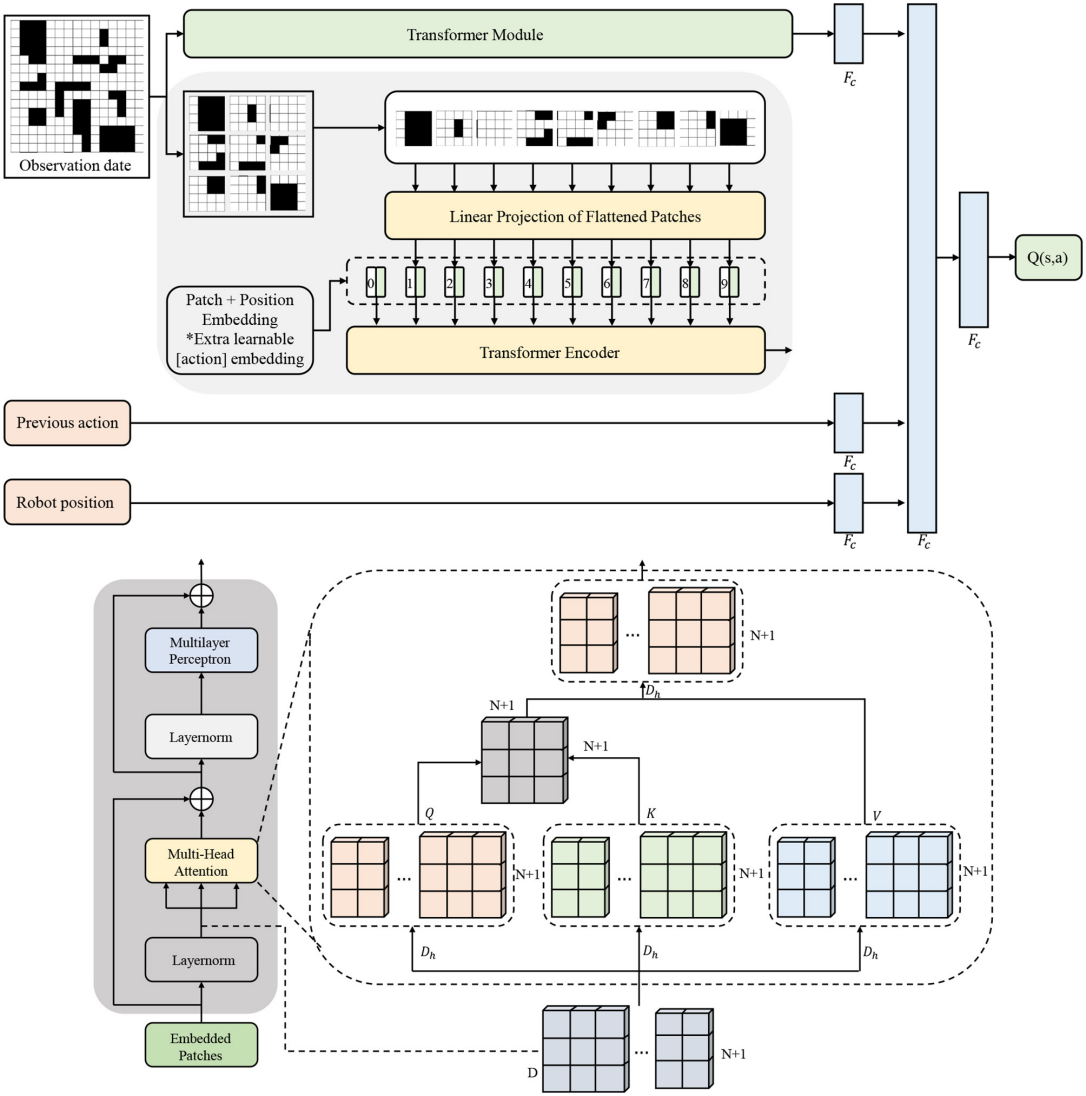
Transformer architecture diagram.

To achieve efficient and intelligent obstacle avoidance for mobile robots in complex and dynamic environments, we adopt the Deep Q-Network (DQN) as the core policy learning algorithm. On this basis, a spatiotemporal perception mechanism is integrated to construct an intelligent obstacle avoidance system that unifies perception and decision-making. The DQN algorithm approximates the state-action value function through a deep neural network, effectively addressing the limitations of traditional Q-learning in high-dimensional state spaces while offering strong policy learning capabilities and environmental adaptability. Our enhanced DQN architecture integrating spatiotemporal Transformer features is detailed in Figure 2, where the Transformer module processes multimodal observations into high-dimensional representations that enable the Q-network to make globally-informed decisions.

In our approach, the observation space S encompasses multi-modal sensory inputs including RGB images, depth maps, and robot kinematic states. Specifically, each state $s_{t}\in S$ is defined as $s_{t}=\left\{I_{t}^{\text{rgb}},I_{t}^{\text{depth}},p_{t},v_{t},\theta_{t}\right\}$, where $I_{t}^{\text{rgb}}\in {\mathbb{R}}^{H\times W\times 3}$ represents the RGB observation, $I_{t}^{\text{depth}}\in {\mathbb{R}}^{H\times W\times 1}$ denotes the depth information, and {*p*_*t*_, *v*_*t*_, θ_*t*_} capture the robot's position, velocity, and orientation respectively.

As illustrated in Figure 2, our architecture processes multimodal inputs through the Transformer module to generate embedded patches, which are then fed into the modified Q-network. The patch + position embedding corresponds to our spatiotemporal feature integration, while the multi-head attention mechanism enables selective focus on critical obstacles. The final *Q*(*s, a*) output directly maps to discrete navigation actions.

Within the reinforcement learning framework, the robot obstacle avoidance task can be modeled as a Markov Decision Process (MDP), denoted as a quintuple:


(1) $$\begin{array}{l} M=\langle S,A,P,R,\gamma \rangle \end{array}$$

where S represents our enhanced state space that integrates spatiotemporal features extracted by the Transformer encoder, specifically $S=\left\{Z^{\prime \prime },p_{t},v_{t},\theta_{t}\right\}$ where *Z*″ denotes the Transformer output features and {*p*_*t*_, *v*_*t*_, θ_*t*_} represent robot kinematic states; A denotes our discrete action space A={forward,left,right,stop} designed for safe navigation in dynamic environments. *P*(*s*′|*s, a*) is the state transition probability, describing the probability of transitioning to the next state *s*′ after executing action *a* in state *s*; *R*(*s, a*) is the reward function, defining the feedback obtained after performing an action; γ ∈ [0, 1] is the discount factor, used to balance long-term returns and immediate rewards.

To handle the high-dimensional observation space effectively, we employ a hierarchical feature extraction strategy where convolutional layers process visual inputs while fully connected layers handle kinematic states. The spatiotemporal attention mechanism (detailed in Section 3.2) then selects the most relevant features across both spatial and temporal dimensions for decision-making.

In practice, our adapted DQN processes the Transformer output features Z (from Equation 24) as state representations, enabling the Q-network to make decisions based on globally-aware spatiotemporal features rather than local observations. The training process incorporates curriculum learning where obstacle complexity gradually increases, and the replay buffer maintains temporal windows to preserve sequential dependencies essential for dynamic obstacle prediction

In DQN, a deep neural network *Q*_θ_(*s, a*) is employed to approximate the Q-function, where θ denotes the network parameters. The network takes the current state (e.g., image) as input and outputs Q-value estimates for each possible action. Training is performed by minimizing the mean squared error loss function:


(2) $$\begin{array}{l} L\left(\theta \right)={\text{𝔼}}_{\left(s,a,r,s^{\prime }\right)\sim D}\left[{\left(y_{t}-Q_{\theta }\left(s,a\right)\right)}^{2}\right] \end{array}$$

where the target Q-value *y*_*t*_ is defined as:


(3) $$\begin{array}{l} y_{t}=r+\gamma \underset{a^{\prime }}{\max}Q_{\theta^{-}}\left(s^{\prime },a^{\prime }\right) \end{array}$$

Here, θ^−^ represents the parameters of a target network, which are periodically copied from the current network parameters θ to stabilize training.

To further improve the stability of value estimation and the convergence speed of the policy, we adopt a decoupled action selection and evaluation process. The target value is modified as follows:


(4) $$\begin{array}{l} y_{t}=r+\gamma Q_{\theta^{-}}\left(s^{\prime },\arg\underset{a^{\prime }}{\max}Q_{\theta }\left(s^{\prime },a^{\prime }\right)\right) \end{array}$$

Our reward function *R*(*s, a*) is designed to encourage safe navigation while maintaining efficiency:


(5) $$\begin{array}{l} R\left(s,a\right)=R_{\text{goal}}+R_{\text{collision}}+R_{\text{progress}}+R_{\text{smoothness}} \end{array}$$

where *R*_goal_ provides positive reward for reaching the target, *R*_collision_ heavily penalizes collisions (−100), *R*_progress_ rewards forward movement toward the goal, and *R*_smoothness_ encourages stable control actions to avoid oscillatory behavior.

Additionally, considering that different actions do not always have significant differences in certain states, we decompose the Q-value function into a state-value function *V*(*s*) and an advantage function *A*(*s, a*), formulated as:


(6) $$\begin{array}{l} Q\left(s,a;\theta ,\alpha ,\beta \right)=V\left(s;\theta ,\beta \right)\\ \;+\left(A\left(s,a;\theta ,\alpha \right)-\frac{1}{\left|A\right|}\underset{a^{\prime }}{\sum }A\left(s,a^{\prime };\theta ,\alpha \right)\right) \end{array}$$

In this formulation, θ represents the shared parameters of the convolutional layers; α corresponds to the parameters of the advantage function branch; β corresponds to the parameters of the state-value function branch.

The DQN framework follows standard implementation with experience replay and target networks. We adopt Double DQN and Dueling DQN variants to improve learning stability and value estimation accuracy.

### 3.2 Spatiotemporal attention mechanism

**Figure 3:**
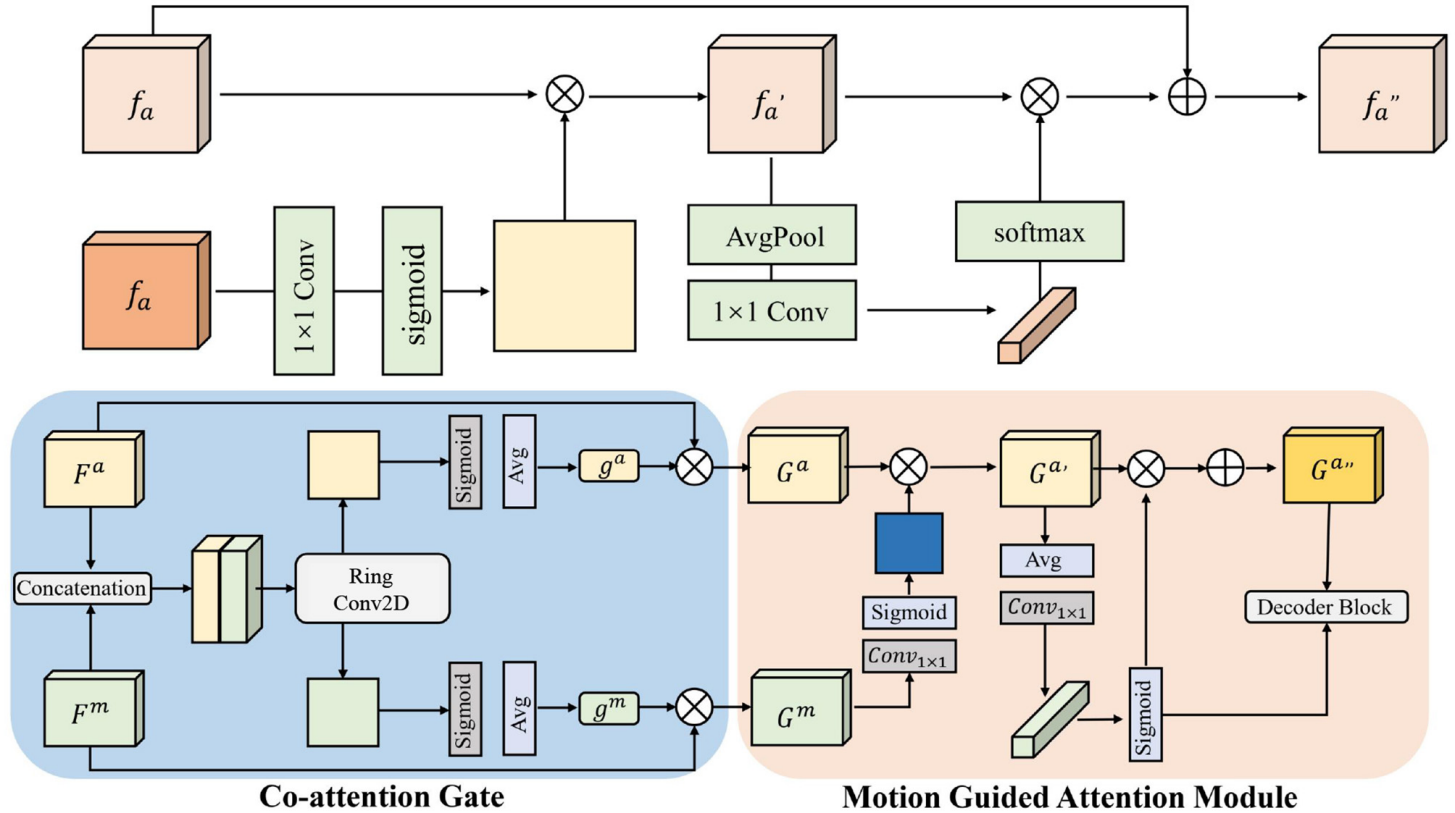
Architecture diagram of SPA.

In the end-to-end intelligent obstacle avoidance method proposed in this paper, a spatiotemporal attention mechanism is introduced as a key module to enhance the robot's perception of the temporal evolution and spatial distribution of obstacles in dynamic environments. Unlike traditional static image-based perception, the information received by a robot during real-world obstacle avoidance is characterized by significant temporal dependencies and spatial locality. Changes in obstacle positions, motion trends, and occlusion relationships often require joint modeling of both temporal and spatial dimensions. To address this, we design a spatiotemporal fusion model based on the Self-Attention mechanism, which extracts critical dynamic behavioral features from sequential historical states and models both local structures and global dependencies in the spatial dimension, thereby comprehensively improving the robot's understanding of complex scenarios and the robustness of its decision-making policy. The architecture is shown in Figure 3.

Figure 3 illustrates the detailed architecture of our Spatiotemporal Attention (SPA) mechanism, which consists of three main components: Co-attention Gate, Motion Guided Attention Module, and Decoder Block. The Co-attention Gate takes feature maps *F*^*a*^ and *F*^*m*^ as inputs, where *F*^*a*^ represents appearance features and *F*^*m*^ denotes motion features extracted from consecutive frames. The gate mechanism uses average pooling and 1 × 1 convolutions followed by sigmoid activation to generate attention weights *g*^*a*^ and *g*^*m*^, which modulate the importance of appearance and motion information respectively. The Motion Guided Attention Module further refines these features through ring convolution operations and sigmoid gating to produce enhanced spatial-temporal representations *G*^*a*^ and *G*^*m*^. Finally, the Decoder Block integrates these multi-scale features and outputs the final attended representation *f*^a*^ that captures both spatial dependencies and temporal dynamics essential for obstacle avoidance decision-making.

At each time step *t*, the robot obtains a sequence of continuous observation image frames of length *T* through its sensors, denoted as:


(7) $$\begin{array}{l} X=\left\{x_{t-T+1},x_{t-T+2},\ldots ,x_{t}\right\},x_{i}\in {\mathbb{R}}^{H\times W\times C} \end{array}$$

where *H*, *W*, and *C* represent the height, width, and number of channels of each image, respectively. For unified processing, each image frame is first embedded into a fixed-dimensional feature vector through convolutional layers or an encoder:


(8) $$\begin{array}{l} F=\left\{f^{a},f^{m}\right\} \end{array}$$

where *f*^*a*^ represents appearance features and *f*^*m*^ denotes motion features as shown in Figure 3, with each feature vector $f_{i}\in {\mathbb{R}}^{d}$

The feature sequence is then input into the spatiotemporal attention module for processing. To jointly model temporal dependencies and spatial perception, a dual-attention mechanism is introduced, consisting of a Temporal Attention module and a Spatial Attention module, defined as follows.

Temporal attention aims to capture key temporal features from the historical state sequence that influence current decision-making. For each time step *i* ∈ [*t*−*T*+1, *t*], query, key, and value vectors are obtained through linear transformations:

Following the Co-attention Gate module in Figure 3, we compute gated features:


(9) $$\begin{array}{ll} g^{a} & =\sigma \left({\text{Conv}}_{1\times 1}\left(\text{AvgPool}\left(f^{a}\right)\right)\right)⊙f^{a} \end{array}$$

(10) $$\begin{array}{ll} g^{m} & =\sigma \left({\text{Conv}}_{1\times 1}\left(f^{m}\right)\right)⊙f^{m} \end{array}$$

All time steps are then organized into matrix form:


(11) $$\begin{array}{l} Q_{T}={\left[q_{t-T+1},\ldots ,q_{t}\right]}^{T},\;K_{T}={\left[k_{t-T+1},\ldots ,k_{t}\right]}^{T},\\ V_{T}={\left[v_{t-T+1},\ldots ,v_{t}\right]}^{T} \end{array}$$

The temporal attention output is computed as:


(12) $$\begin{array}{l} {\text{Attn}}_{time}\left(F\right)=\text{softmax}\left(\frac{Q_{T}K_{T}^{T}}{\sqrt{d_{k}}}\right)V_{T} \end{array}$$

In our formulation, *f*^*a*^ represents appearance features, *f*^*m*^ denotes motion features, *G*^*a*^ and *G*^*m*^ are the corresponding gated attention maps, and the operations ⊙ and ⊕ represent element-wise multiplication and addition respectively. This process achieves weighted aggregation of key historical moments, strengthening the robot's understanding of obstacle motion trends and behavior patterns.

For spatial attention modeling, at each time step *i*, based on the image feature map $f_{i}\in {\mathbb{R}}^{H\times W\times d}$, positional embeddings and local window partitioning are applied to extract spatial information. Denoting the embedding at each spatial position as $f_{i}^{\left(h,w\right)}\in {\mathbb{R}}^{d}$, the attention computation is as follows:


(13) $$\begin{array}{l} q_{h,w}=W_{Q}^{\left(s\right)}f_{i}^{\left(h,w\right)},\;k_{h^{\prime },w^{\prime }}=W_{K}^{\left(s\right)}f_{i}^{\left(h^{\prime },w^{\prime }\right)},\;v_{h^{\prime },w^{\prime }}=W_{V}^{\left(s\right)}f_{i}^{\left(h^{\prime },w^{\prime }\right)} \end{array}$$

The corresponding spatial attention output is:


(14) $$\begin{array}{l} {\text{Attn}}_{space}{\left(f_{i}\right)}_{h,w}=\underset{\left(h^{\prime },w^{\prime }\right)\in N\left(h,w\right)}{\sum }\alpha_{\left(h,w\right),\left(h^{\prime },w^{\prime }\right)}\cdot v_{h^{\prime },w^{\prime }} \end{array}$$

with the attention weight computed by:


(15) $$\begin{array}{l} \alpha_{\left(h,w\right),\left(h^{\prime },w^{\prime }\right)}=\frac{\exp\left(q_{h,w}\cdot k_{h^{\prime },w^{\prime }}/\sqrt{d_{k}}\right)}{\sum_{\left(h^{\prime \prime },w^{\prime \prime }\right)\in N\left(h,w\right)}\exp\left(q_{h,w}\cdot k_{h^{\prime \prime },w^{\prime \prime }}/\sqrt{d_{k}}\right)} \end{array}$$

where N(h,w) denotes the neighborhood region centered at (*h, w*).

After obtaining the temporal attention output ${\hat{F}}_{i}={\text{Attn}}_{time}\left(F\right)$ and the spatial attention output ${\hat{f}}_{i}^{space}$ for each frame, a spatiotemporal fusion module is introduced to aggregate the spatial features of key frames in the time series:


(16) $$\begin{array}{l} {\tilde{f}}_{t}=\overset{t}{\underset{i=t-T+1}{\sum }}{\text{λ}}_{i}\cdot \text{Flatten}\left({\hat{f}}_{i}^{space}\right),\;{\text{λ}}_{i}=\text{softmax}\left(\phi \left({\hat{f}}_{i}\right)\right) \end{array}$$

where ϕ(·) is an attention scoring function used to compute fusion weights. The final fused feature ${\tilde{f}}_{t}$ is then fed into the policy network or reinforcement learning decision module.

In summary, the spatiotemporal attention mechanism introduced in this paper provides essential perception and modeling support for intelligent obstacle avoidance by mobile robots in complex dynamic environments. By jointly modeling the behavioral evolution features in the temporal dimension and obstacle distribution information in the spatial dimension, it addresses the limitations of traditional perception methods in terms of locality, sequential dependency modeling, and global consistency representation. Temporal attention dynamically captures critical historical states, revealing obstacle movement trends and potential risk changes, while spatial attention precisely focuses on key local regions in the current observation frame, enabling fine-grained recognition of multiple obstacles and multi-scale targets. The combination of the two not only enhances the robot's overall environmental understanding but also provides higher-quality, decision-relevant semantic feature inputs for downstream policy networks, thereby significantly improving the robustness and generalization capability of obstacle avoidance strategies in complex, changing scenarios. Theoretically, this mechanism exhibits excellent scalability and modularity, allowing it to naturally integrate with deep reinforcement learning frameworks and build a unified end-to-end perception-decision system. It thus lays a solid foundation for the development of high-performance, adaptive robotic navigation systems.

### 3.3 Transformer architecture

**Figure 4:**
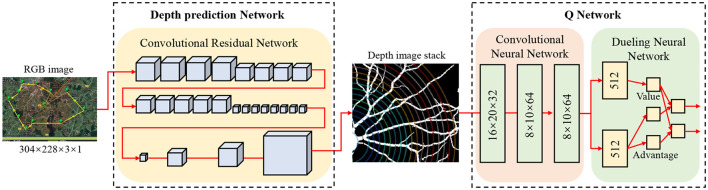
Architecture diagram of DQN.

The key distinction of our “end-to-end” transformer lies in its architectural adaptations for direct sensor-to-action mapping: (1) Input Integration: Unlike conventional transformers that process single-modality sequential data, our architecture simultaneously handles RGB images, depth information, and kinematic states through unified patch embeddings, (2) Attention Mechanism: We modify the self-attention to incorporate spatial proximity bias for obstacle-aware feature selection, (3) Output Decoder: The final layer directly outputs Q-values for discrete actions rather than requiring separate decision-making modules, and (4) Training Objective: The entire pipeline is optimized end-to-end using reinforcement learning signals rather than supervised learning on intermediate representations. As the core module for perception and modeling, the Transformer architecture is employed to uniformly process sequential input data from multimodal sensors. With its powerful global modeling capability and multi-head self-attention mechanism, it effectively extracts key spatiotemporal features that influence robot obstacle avoidance behavior. Compared to traditional Convolutional Neural Networks (CNNs) or Recurrent Neural Networks (RNNs), the Transformer architecture can model long-term dependencies in parallel and maintains strong feature alignment capabilities in both spatial and temporal dimensions, making it particularly well-suited for obstacle behavior modeling and multisource information fusion in dynamic environments. The architecture is shown in Figure 4.

Within a temporal window of length *T*, the robot collects a sequence of multimodal perception frames:


(17) $$\begin{array}{l} X=\left\{x_{1},x_{2},\ldots ,x_{T}\right\},x_{t}\in {\mathbb{R}}^{H\times W\times C} \end{array}$$

where each *x*_*t*_ represents the image/depth frame obtained at time *t*, and *H*, *W*, and *C* denote the height, width, and number of channels of the frame, respectively. Each frame is first subjected to feature extraction and flattening, followed by a linear transformation to obtain a unified-dimensional embedding representation:


(18) $$\begin{array}{l} z_{t}=\text{Flatten}\left(x_{t}\right)\cdot W_{E}+b_{E},z_{t}\in {\mathbb{R}}^{d} \end{array}$$

where $W_{E}\in {\mathbb{R}}^{\left(H\times W\times C\right)\times d}$ is the embedding matrix and $b_{E}\in {\mathbb{R}}^{d}$ is the bias vector. To clarify the architectural integration: the ResNet-based feature extractor (Figure 4) serves as the visual encoder that processes raw sensor inputs into compact spatial representations $z_{t}\in {\mathbb{R}}^{512}$. These features are then temporally organized and fed into our Transformer encoder (described below) to capture spatiotemporal dependencies. The Transformer output *Z* is subsequently processed by the Dueling DQN network (also shown in Figure 4) to generate action decisions. To preserve temporal information, a positional encoding $p_{t}\in {\mathbb{R}}^{d}$ is introduced, and the final input sequence to the Transformer is:


(19) $$\begin{array}{l} Z=\left\{z_{1}+p_{1},z_{2}+p_{2},\ldots ,z_{T}+p_{T}\right\} \end{array}$$

The Transformer encoder consists of multiple stacked layers, each composed of a Multi-Head Self-Attention mechanism and a Feed Forward Network (FFN). The core computation within each layer involves three main steps:

For each layer input *Z* = {*z*_1_, *z*_2_, …, *z*_*T*_}, query, key, and value vectors are generated through linear projections:


(20) $$\begin{array}{l} Q=ZW^{Q},\;K=ZW^{K},\;V=ZW^{V},W^{Q},W^{K},W^{V}\in {\mathbb{R}}^{d\times d_{k}} \end{array}$$

The attention matrix is then computed as:


(21) $$\begin{array}{l} \text{Attention}\left(Q,K,V\right)=\text{softmax}\left(\frac{QK^{T}}{\sqrt{d_{k}}}\right)V \end{array}$$

To enhance the model's representation capability, *h* attention heads are introduced, with each head corresponding to different linear projections:


(22) $$\begin{array}{l} \text{MultiHead}\left(Z\right)=\text{Concat}\left({\text{head}}_{1},\ldots ,{\text{head}}_{h}\right)W^{O} \end{array}$$

where:


(23) $$\begin{array}{l} {\text{head}}_{i}=\text{Attention}\left(ZW_{i}^{Q},ZW_{i}^{K},ZW_{i}^{V}\right),W^{O}\in {\mathbb{R}}^{hd_{v}\times d} \end{array}$$

After each sublayer, residual connections and Layer Normalization are applied:


(24) $$\begin{array}{l} Z^{\prime }=\text{LayerNorm}\left(Z+\text{MultiHead}\left(Z\right)\right) \end{array}$$

(25) $$\begin{array}{l} Z^{\prime \prime }=\text{LayerNorm}\left(Z^{\prime }+\text{FFN}\left(Z^{\prime }\right)\right) \end{array}$$

The Feed Forward Network (FFN) is defined as:


(26) $$\begin{array}{l} \text{FFN}\left(x\right)=\sigma \left(xW_{1}+b_{1}\right)W_{2}+b_{2} \end{array}$$

where $W_{1}\in {\mathbb{R}}^{d\times d_{ff}},W_{2}\in {\mathbb{R}}^{d_{ff}\times d}$.

To further enhance the Transformer's ability to model temporal continuity and spatial distribution characteristics, this paper introduces a position-sensitive spatiotemporal weighting mask mechanism within the multi-head attention module. During the attention weight computation, positions with larger spatial or temporal distances are assigned lower weights:


(27) $$\begin{array}{l} \alpha_{ij}=\frac{\exp\left(\frac{q_{i}\cdot k_{j}}{\sqrt{d_{k}}}-\text{λ}\cdot \delta_{ij}\right)}{\sum_{j^{\prime }}\exp\left(\frac{q_{i}\cdot k_{j^{\prime }}}{\sqrt{d_{k}}}-\text{λ}\cdot \delta_{ij^{\prime }}\right)} \end{array}$$

where δ_*ij*_ represents the spatiotemporal distance between positions *i* and *j*, and λ is a decay factor used to suppress interference from distant positions. In the final encoded output *Z*″, each vector carries rich semantic information from multiple time steps and spatial locations, serving as the state input to the reinforcement learning policy network to guide the robot's obstacle avoidance decision-making.

This contrasts sharply with traditional robotic systems that employ separate modules for perception (CNN-based feature extraction), mapping (SLAM), planning (A* or RRT), and control (PID controllers), each requiring manual tuning and intermediate data conversion. Our end-to-end approach eliminates these boundaries by learning a unified representation that directly maps sensory observations to optimal actions through the transformer's global attention mechanism.

In summary, as a sequence modeling method based on the self-attention mechanism, the Transformer architecture offers an efficient, unified perception and decision-making modeling paradigm for robot obstacle avoidance tasks through its exceptional global dependency modeling capability and parallel computing advantages. By dynamically assigning weights to relationships between time steps and spatial positions in the input sequence, the Transformer adaptively focuses on the most critical spatiotemporal feature information for obstacle avoidance decision-making, significantly enhancing the robot's understanding of dynamic environments and response speed. By integrating the Transformer architecture into the reinforcement learning framework, this study constructs an end-to-end unified learning pipeline from raw perception to behavioral decision-making. This approach also provides a theoretically rigorous and practically efficient solution for multimodal information fusion, multi-object dependency modeling, and decision policy optimization in complex environments. Therefore, the Transformer is not only a perception enhancement module but also a fundamental component for improving the synergy, generalization, and robustness of autonomous robot obstacle avoidance systems.

## 4 Experiment

### 4.1 Experimental setup and dataset

To verify the effectiveness and generalization capability of the proposed method, all experiments were conducted on a unified software and hardware platform to ensure the comparability and reproducibility of different approaches under the same environment. All models were implemented based on PyTorch and trained on a high-performance workstation running Ubuntu 20.04, equipped with an NVIDIA GeForce RTX 4090 GPU (24 GB VRAM) and an Intel Core i9-13900K processor. For model training, the Adam optimizer was employed to update the parameters of the policy network, with an initial learning rate set to 1e-4 and a batch size of 64. The target network was synchronized every 1,000 steps. The training process consisted of 1,000 epochs, and a dropout rate of 0.1 was applied to prevent overfitting.

This work systematically conducted experiments and performance evaluations of the proposed end-to-end robot intelligent obstacle avoidance method using three interactive simulation environments (RoboTHOR, CARLA, TurtleBot3) for data generation and one real-world dataset (EuRoC MAV) for validation. These cover a wide range of application scenarios, from static indoor environments to dynamic outdoor scenes, and from ground robots to aerial platforms, thereby verifying the model's generalization ability and robustness under multi-modal input and complex spatiotemporal conditions. To clarify our experimental methodology: RoboTHOR, CARLA, and TurtleBot3 serve as simulation environments where we generate synthetic training and testing data by running our proposed algorithm in various scenarios. The EuRoC MAV represents a real-world dataset containing pre-recorded multimodal sensor data from actual aerial vehicle flights. This hybrid approach allows us to evaluate our method's performance across both controlled simulation conditions and real-world data complexities. The four datasets used are introduced as follows:

- RoboTHOR

Developed by the Allen Institute for AI, RoboTHOR (Deitke et al., 2020) is an interactive visual navigation and obstacle avoidance simulation platform that provides highly realistic 3D indoor home environments. It supports robots performing navigation and obstacle avoidance tasks in multi-room structures. The platform integrates various sensory inputs (such as RGB images, depth maps, and semantic labels) and control outputs, making it suitable for training and evaluating end-to-end vision-control integrated models. In this study, a multi-scene indoor obstacle avoidance test set was constructed using RoboTHOR to evaluate the path planning accuracy and local obstacle avoidance capability of the proposed method in static and mildly dynamic indoor environments.

- CARLA

CARLA (Gannamaneni et al., 2021) is an open-source autonomous driving simulator built on Unreal Engine, widely used for research on navigation and obstacle avoidance in urban road environments. It provides a realistic simulation of dynamic elements such as traffic flow, traffic lights, pedestrians, and vehicles, and supports multi-sensor simulation (RGB, LiDAR, IMU, GPS, etc.). In this study, CARLA was employed to build highly dynamic complex obstacle scenarios to examine the model's spatiotemporal modeling ability and obstacle avoidance robustness in urban environments with dynamic obstacles and sudden disturbances.

- TurtleBot3

TurtleBot3 (Kashyap and Konathalapalli, 2025) is a compact mobile robot platform officially supported by ROS, whose Gazebo simulation model is commonly used for teaching and research on tasks such as path planning, navigation, and obstacle avoidance. Its clean and controllable simulation environment is ideal for training and debugging reinforcement learning models. In this work, the proposed method was trained and evaluated across multiple TurtleBot3 indoor maps. Taking advantage of its precise control model and high reproducibility, the algorithm's path efficiency and control stability in typical indoor environments were verified.

- EuRoC MAV Dataset

The EuRoC MAV Dataset (Burghoffer et al., 2023) is a multi-modal navigation dataset for Micro Aerial Vehicles (MAVs), provided by the European Robotics Research Center. It includes real aerial photography data from multiple industrial and indoor scenarios, combining high-frequency image frames, IMU sensor data, and ground-truth pose labels. Widely used in tasks such as VIO, SLAM, and obstacle avoidance, this study selected several challenging scenes from this dataset to test the obstacle avoidance decision performance of the proposed method on aerial platforms, with particular focus on strategy adaptability and sequential modeling ability under highly dynamic, high-degree-of-freedom movements.

### 4.2 Evaluation metrics

To comprehensively assess the performance of the proposed deep reinforcement learning-based intelligent obstacle avoidance method incorporating a spatiotemporal Transformer architecture, four key evaluation metrics were selected from multiple dimensions, including obstacle avoidance capability, path efficiency, and task response time. These metrics effectively reflect the safety, intelligence, and practicality of the model in different environments and are suitable for obstacle avoidance tasks in dynamic and complex scenarios.

Our comprehensive hyperparameter configuration includes DQN parameters with learning rate α = 1e − 4 (selected from tested range 1e − 5 to 1e − 3), discount factor γ = 0.99, experience replay buffer size of 100, 000, target network updates every 1, 000 steps, and ε-greedy exploration decaying from 1.0 to 0.01 over 50, 000 steps; Transformer architecture with hidden dimension *d* = 512, 8 attention heads, 6 encoder layers, feedforward dimension *d*_ff_ = 2048, dropout rate of 0.1, and maximum positional encoding length of 1, 000; spatiotemporal attention mechanism using temporal window *T* = 10 frames, 7 × 7 spatial neighborhood, attention temperature τ = 0.1, and fusion weight decay λ = 0.001; and training configuration employing batch size 64, Adam optimizer (β_1_ = 0.9, β_2_ = 0.999), weight decay 1e − 4, gradient clipping threshold 1.0, and exponential learning rate decay of 0.95 every 100 epochs. Systematic sensitivity analysis across all parameters revealed robust performance within ±20% hyperparameter variations, with learning rate and attention head number identified as the most critical factors affecting convergence speed and final performance respectively.

- Obstacle Avoidance Success Rate (OASR)

This metric measures whether the robot successfully avoids all obstacles and reaches the target point during the task. It reflects the overall obstacle avoidance capability and strategy effectiveness of the system.


(28) $$\begin{array}{l} Success\;Rate\left(OASR\right)=\frac{N_{success}}{N_{total}}\times 100\% \end{array}$$

where *N*_*success*_ is the number of successful, collision-free task completions, and *N*_*total*_ is the total number of experiments. In this work, OASR is used to compare the safety navigation capability of different methods in complex dynamic environments and serves as a core metric for strategy reliability evaluation.

- Collision Rate (CR)

The Collision Rate measures the frequency of collisions occurring during the navigation process, serving as a negative indicator for obstacle avoidance robustness and safety. It is defined as:


(29) $$\begin{array}{l} Collision\;Rate\left(CR\right)=\frac{N_{collision}}{N_{total}}\times 100\% \end{array}$$

where *N*_*collision*_ is the number of tasks in which at least one collision occurred. The proposed strategy enhances the perception of dynamic obstacles through the spatiotemporal modeling capability of the Transformer architecture, thereby reducing collision rates at the control level and improving system safety.

- Average Path Length

This metric reflects the spatial efficiency of the path chosen by the robot from the starting point to the target point. Shorter paths generally indicate more optimized strategies.


(30) $$\begin{array}{l} Average\;Path\;Length=\frac{1}{N_{success}}\overset{N_{success}}{\underset{i=1}{\sum }}L_{i} \end{array}$$

where *L*_*i*_ represents the total path length in the *i*^*th*^ successful navigation, and *N*_*success*_ is the number of successful task completions. In this work, this metric is used to compare the global efficiency of different strategies in path planning, with particular attention to path convergence performance under dynamic obstacle interference.

- Average Navigation Time

The Average Navigation Time assesses the robot's response speed and execution efficiency in completing the task, which is closely related to control decision latency and the stability of obstacle avoidance behavior.


(31) $$\begin{array}{l} Average\;Navigation\;Time=\frac{1}{N_{success}}\overset{N_{success}}{\underset{i=1}{\sum }}T_{i} \end{array}$$

where *T*_*i*_ represents the time taken to successfully complete the *i*^*th*^ task, and *N*_*success*_ is the number of successful task completions. The proposed end-to-end architecture reduces intermediate processing time from perception to control and improves the strategy's foresight through the global modeling capability of the Transformer, effectively shortening the task response time.

### 4.3 Experimental results analysis

We compare the proposed algorithm with representative baseline methods that encompass different paradigms in the field of obstacle avoidance based on deep reinforcement learning. A bio-inspired multi-underwater spherical robot control system employs a mobile obstacle avoidance strategy using a collaborative formation mode. This method combines a perception module based on convolutional neural networks (CNN) with standard deep reinforcement learning (DQN) for multi-robot coordination, representing a traditional vision-based reinforcement learning approach. A fast finite-time binary formation control method with obstacle avoidance functionality, suitable for multi-agent systems with delays. This method employs a distributed consensus algorithm combined with A3C reinforcement learning, focusing on multi-agent coordination in delayed environments. Baseline methods are implemented using their original hyperparameters (if available) or carefully tuned to ensure optimal performance in the experimental environment.

To comprehensively verify the effectiveness of the proposed end-to-end robot intelligent obstacle avoidance method based on deep reinforcement learning and a spatiotemporal Transformer architecture, this section systematically analyzes and compares the experimental results on four different types of datasets (RoboTHOR, CARLA, TurtleBot3, EuRoC MAV). We evaluate the model's performance under multi-scene and multi-task conditions from four key metrics: obstacle avoidance success rate (OASR), collision rate (CR), average path length (APL), and average navigation time (ANT). Particular emphasis is placed on analyzing the model's obstacle avoidance robustness, path planning efficiency, and real-time control capability in complex dynamic environments. Additionally, comparisons with various representative baseline models are conducted to highlight the advantages of the proposed method in global modeling, spatiotemporal feature extraction, and policy optimization.

As shown in Table 1, in the RoboTHOR environment, the proposed method achieves an OASR of 91.21%, significantly higher than the best-performing comparison method (MOAC-MURS, 88.15%) and over 16 percentage points better than the lowest value (Hu et al., 75.08%). Meanwhile, the CR of the proposed method is only 3.15%, much lower than other methods (e.g., DWA-DRL. 20.25%, PMO-DQN. 21.53%), demonstrating strong policy robustness and safety. In terms of path efficiency, the APL is only 16.85 meters, superior to all comparison models, indicating that the model can seek more optimal strategies while ensuring safety. The proposed method also excels in ANT, requiring only 46.82 s to complete a task, significantly faster than FTBF-OA. (68.74 s) and Hu et al. (69.99 s), demonstrating quicker response capability and control efficiency. In the more challenging CARLA urban driving environment, the proposed method continues to maintain its leading performance, achieving an OASR of 92.35%, the only model exceeding 90%, far surpassing comparison methods such as PMO-DQN. (74.96%) and CBF-MPC. (75.01%). In terms of CR, it remains the lowest at 4.2%, highlighting excellent dynamic obstacle avoidance capability. For path planning and time efficiency, the proposed method requires only 18.2 meters and 48.13 s, outperforming other methods like PMO-DQN. (47.98 meters, 70.08 s) by a wide margin.

**Table 1:** Comparison of indicators of various models on RoboTHOR and CARLA dataset.

**Method**	**RoboTHOR**				**CARLA**			
	**OASR(%)**	**CR(%)**	**APL(m)**	**ANT(s)**	**OASR(%)**	**CR(%)**	**APL(m)**	**ANT(s)**
MOAC-MURS (Li et al., 2025a)	88.15	10.12	22.34	52.36	80.56	17.23	36.8	63.65
DWA-DRL (Wang et al., 2025a)	77.23	20.25	38.4	65.93	83.89	5.57	24.55	54.56
FTBF-OA (Chang et al., 2025)	76.12	14.98	45.2	68.74	81.67	15.05	33.65	66.87
CBF-MPC (Liu et al., 2025)	79.45	11.34	32.15	62.41	75.01	22.75	41.3	67.38
PMO-DQN (Hu et al., 2025)	75.08	21.53	49.75	69.99	74.96	24.03	47.98	70.08
GP-RL-UAV (Enriquez et al., 2025)	82.34	12.56	27.91	57.15	82.78	13.78	29.1	58.1
Ours	91.21	3.15	16.85	46.82	92.35	4.2	18.2	48.13

**Figure 5:**
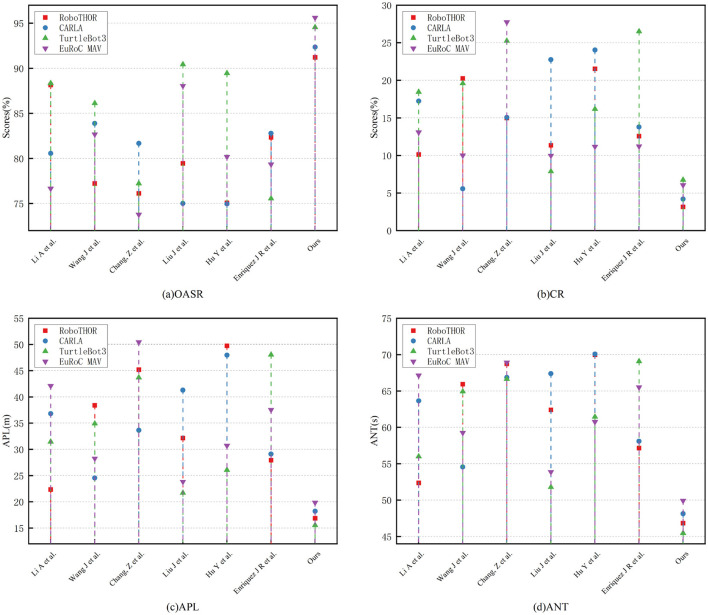
Comparative visualization of each model indicator on four dataset.

In summary, in Table 2 and Figure 5, experimental results on TurtleBot3 and EuRoC MAV further confirm the universality and adaptability of the proposed method in static + dynamic, ground + aerial, structured + unstructured environments. Whether in OASR, path efficiency, or task response time, the proposed method consistently demonstrates significant advantages, showcasing the natural strength of the Transformer in spatiotemporal feature modeling and the potential of deep reinforcement learning for policy adaptive optimization. It also indicates that the proposed perception-decision integrated architecture has broad practical deployment value.

**Table 2:** Comparison of indicators of various models on urtleBot3 and EuRoC MAV dataset.

**Method**	**TurtleBot3**				**EuRoC MAV**			
	**OASR(%)**	**CR(%)**	**APL(m)**	**ANT(s)**	**OASR(%)**	**CR(%)**	**APL(m)**	**ANT(s)**
MOAC-MURS (Li et al., 2025a)	88.34	18.45	31.46	56	76.66	13.08	42.11	67.15
DWA-DRL (Wang et al., 2025a)	86.12	19.6	34.92	64.93	82.67	10.01	28.25	59.25
FTBF-OA (Chang et al., 2025)	77.23	25.25	43.69	66.64	73.78	27.73	50.46	68.93
CBF-MPC (Liu et al., 2025)	90.43	7.89	21.71	51.79	88.05	9.98	23.82	53.87
PMO-DQN (Hu et al., 2025)	89.45	16.17	26.07	61.45	80.19	11.17	30.71	60.76
GP-RL-UAV (Enriquez et al., 2025)	75.56	26.5	48.05	69.08	79.34	11.22	37.53	65.51
Ours	94.55	6.75	15.52	45.46	95.61	6.05	19.86	49.92

As shown in Table 3, the resource consumption and efficiency comparison experiments reveal that the proposed method not only outperforms other methods in performance metrics but also exhibits significant advantages in computational efficiency, model size, and inference speed, fully demonstrating the model's lightweight design and deployment friendliness. On RoboTHOR, the proposed method achieves the shortest training time (61.34 s), over 38% faster than the slowest (Enriquez et al., 99.81 s), indicating higher efficiency in parameter updates. The inference time per step averages only 92.37 milliseconds, notably lower than Wang et al. (199.83 ms) and Li et al. (178.46 ms), favoring real-time robot responses in practical applications. In terms of computational complexity, the proposed method requires only 15.34 GFLOPs, the lowest among all methods (compared to Enriquez et al. 34.17 G, Li et al. 30.48 G), confirming its deployability on resource-constrained platforms. Furthermore, the model's parameter count is only 142.83 M, the smallest among all methods, reflecting highly optimized network design. On the more complex CARLA dataset, the proposed method maintains its lead, with a training time of 63.27 s, over 15% shorter than most other comparison methods. The inference time averages just 95.12 milliseconds, much faster than Wang et al. (148.73 ms), Li et al. (183.27 ms), and Liu et al. (194.59 ms). In terms of computational complexity, the model requires only 16.78 GFLOPs, significantly lower than Li et al. (33.99 G) and Liu et al. (31.12 G). The parameter count remains the lowest at 156.34 M, verifying the model's balanced design in ensuring performance while minimizing computational overhead.

**Table 3:** Comparison of training indicators on RoboTHOR and CARLA dataset.

**Method**	**RoboTHOR**				**CARLA**			
	**Training time(s)**	**Inference time(ms)**	**Flops(G)**	**Para.(M)**	**Training time(s)**	**Inference time(ms)**	**Flops(G)**	**Para.(M)**
MOAC-MURS (Li et al., 2025a)	87.69	178.46	30.48	317.52	97.52	183.27	33.99	368.42
DWA-DRL (Wang et al., 2025a)	68.97	199.83	26.93	264.16	84.19	148.73	27.84	227.91
FTBF-OA (Chang et al., 2025)	93.28	128.53	21.69	235.49	70.58	132.05	22.05	293.05
CBF-MPC (Liu et al., 2025)	75.42	143.29	23.57	392.71	95.73	194.59	31.12	209.68
PMO-DQN (Hu et al., 2025)	82.13	108.64	17.82	188.27	78.36	165.94	19.21	325.78
GP-RL-UAV (Enriquez et al., 2025)	99.81	156.81	34.17	359.03	89.47	117.88	24.36	399.91
Ours	61.34	92.37	15.34	142.83	63.27	95.12	16.78	156.34

**Figure 6:**
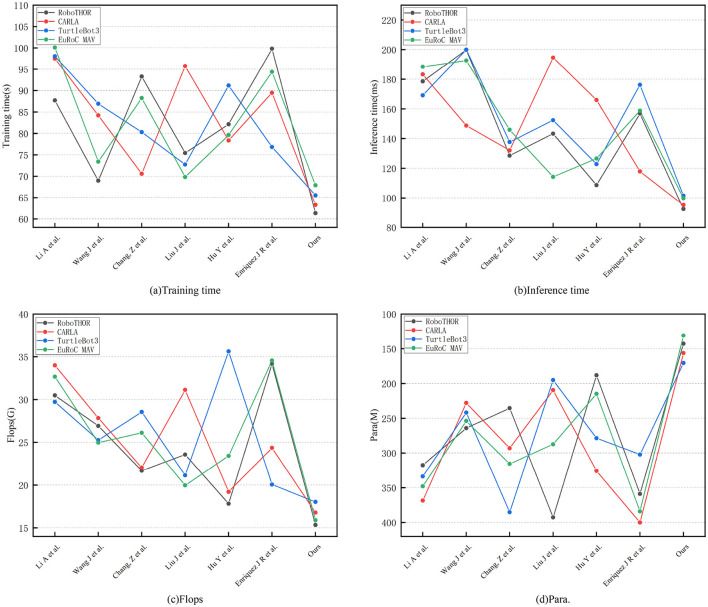
Comparative visualization of training metrics on four datasets.

As shown in Table 4, the proposed method consistently outperforms all comparison methods across all metrics, reflecting a comprehensively optimized approach. On the TurtleBot3 dataset, the training time (65.53 s) is about 10% shorter than the next-best method (Liu et al., 72.76 s), inference time (101.46 milliseconds) is 17% lower than the best competing method (Hu et al., 122.79 milliseconds), and both GFLOPs (18.03G) and parameter count (170.59 M) are the lowest, demonstrating higher computational efficiency and a higher degree of lightweight design. On the EuRoC MAV dataset, the proposed method again maintains its lead, with the shortest training time (67.89 s), inference time (99.83 milliseconds), and the lowest GFLOPs (15.89 G) and parameter count (131.02 M), reducing overhead by 20–50% and 20–60%, respectively, compared to other methods. Notably, certain methods like Enriquez et al. have parameter counts as high as 384.12 M, yet their inference time (158.77 ms) remains significantly inferior to the proposed method, highlighting the efficiency of the model's structural optimization. Figure 6 presents a comparative visualization of the training metrics for each model in the four datasets. Calculating efficiency relationships and resource utilization patterns is important for practical deployment considerations. We considered the trade-off between efficiency and performance, achieving better results while reducing computational overhead. In terms of scalability across datasets, even with increased environmental complexity, consistent efficiency improvements can be maintained. We highlighted the relationship between model parameters, floating point operations (FLOPs), and actual performance improvements.

**Table 4:** Comparison of training indicators on urtleBot3 and EuRoC MAV dataset.

**Method**	**TurtleBot3**				**EuRoC MAV**			
	**Training time(s)**	**Inference time(ms)**	**Flops(G)**	**Para.(M)**	**Training time(s)**	**Inference time(ms)**	**Flops(G)**	**Para.(M)**
MOAC-MURS (Li et al., 2025a)	98.06	169.08	29.73	333.67	100.08	188.42	32.67	347.98
DWA-DRL (Wang et al., 2025a)	86.92	200.05	25.27	241.73	73.41	192.63	24.98	253.89
FTBF-OA (Chang et al., 2025)	80.29	137.62	28.56	385.24	88.24	145.9	26.11	315.55
CBF-MPC (Liu et al., 2025)	72.76	152.34	21.14	195.12	69.83	114.21	19.97	287.31
PMO-DQN (Hu et al., 2025)	91.17	122.79	35.62	278.49	79.62	126.55	23.42	214.76
GP-RL-UAV (Enriquez et al., 2025)	76.84	176.15	20.06	302.1	94.37	158.77	34.55	384.12
Ours	65.53	101.46	18.03	170.59	67.89	99.83	15.89	131.02

Overall, comprehensive analysis indicates that the proposed method leads not only in obstacle avoidance accuracy and path efficiency but also in model size, computational complexity, training convergence speed, and inference response time. It fully embodies the innovative concept of lightweight, efficient, and deployable model design. Especially in practical robotic applications, the proposed model's high efficiency, low latency, and low resource consumption characteristics provide a solid foundation for deployment on embedded platforms or resource-constrained systems, offering considerable practical value and broad promotion potential.

As shown in Table 5, ablation study results demonstrate that the full model proposed in this paper outperforms all variant models (with any key module removed) on all core metrics across the four datasets, verifying the indispensable roles of the deep reinforcement learning module (DR), spatiotemporal attention mechanism (SPA), and Transformer architecture in the system's overall performance. The full model shows significant advantages in OASR, CR, APL, and ANT, fully reflecting the rationality and synergistic effect of the proposed structural design.

**Table 5:** Ablation experiments of this model on four datasets.

**Module**	**RoboTHOR**				**CARLA**			
	**OASR(%)**	**CR(%)**	**APL(m)**	**ANT(s)**	**OASR(%)**	**CR(%)**	**APL(m)**	**ANT(s)**
w/o DR	73.59	11.34	38.62	76.89	69.42	31.49	41.78	72.16
w/o SPA	61.34	23.78	27.83	67.23	71.83	15.97	32.14	96.08
w/o Trans.	67.28	35.62	51.49	92.54	63.17	26.05	58.36	81.37
Ours	91.21	3.15	16.85	46.82	92.35	4.2	18.2	48.13
**Module**	**TurtleBot3**				**EuRoC MAV**			
	**OASR(%)**	**CR(%)**	**APL(m)**	**ANT(s)**	**OASR(%)**	**CR(%)**	**APL(m)**	**ANT(s)**
w/o DR	66.05	18.23	36.29	79.02	62.78	40.27	34.91	84.65
w/o SPA	64.96	29.16	63.12	69.58	68.53	12.89	44.03	74.93
w/o Trans.	74.31	38.71	29.67	88.41	75.82	22.54	65.59	99.67
Ours	94.55	6.75	15.52	45.46	95.61	6.05	19.86	49.92

On RoboTHOR, the full model achieves an OASR of 91.21%, while removing the DR module drops it to 73.59%, and removing SPA further drops it to 61.34%, demonstrating the crucial role of reinforcement learning for long-term decision-making in dynamic environments and the SPA for feature representation and obstacle perception. Removing the Transformer causes OASR to decline to 67.28% and CR to surge to 35.62%, while APL increases dramatically to 51.49 meters, confirming the importance of Transformer in capturing global temporal dependencies. The full model's APL of 16.85 meters and ANT of 46.82 s remain optimal.

In the CARLA urban scene, the full model maintains its lead with a 92.35% OASR and 4.2% CR. Removing the Transformer drops OASR to 63.17%, increases CR to 26.05%, and significantly raises both APL (58.36 meters) and ANT (81.37 s), showing the critical importance of the Transformer in complex, dynamic, multi-object environments. Removing DR or SPA also results in noticeable performance declines, proving their indispensable roles in policy optimization and spatiotemporal feature extraction.

Similar trends appear on TurtleBot3, where the full model leads with a 94.55% OASR and the lowest CR of 6.75%. Removing SPA causes the OASR to plummet to 64.96%, while removing the Transformer keeps OASR at 74.31% but CR surges to 38.71%, confirming the SPA's role in integrating local and global obstacle information for safe avoidance.

**Figure 7:**
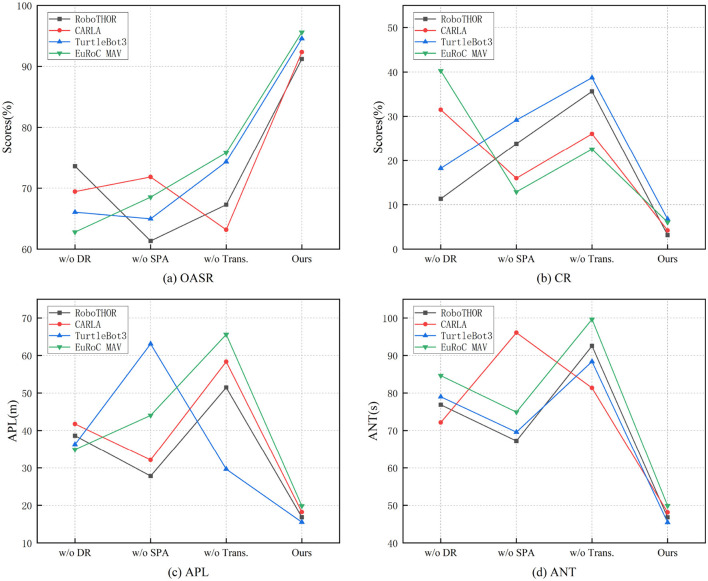
Comparative visualization of ablation experiments on four dataset.

In the EuRoC MAV dataset, the full model achieves a 95.61% OASR and 6.05% CR, proving strong robustness and precise path planning capability in high-DOF, temporally sensitive UAV obstacle avoidance tasks. Removing the Transformer drops OASR to 75.82%, raises CR to 22.54%, and nearly doubles ANT to 99.67 s, demonstrating the Transformer's essential role in modeling high-dimensional state transitions and sequential behaviors. Figure 7 illustrates the results of the ablation experiments. We provide insights into component interdependencies and synergistic effects. The display module provides a hierarchy of the most significant performance improvements contributed by components, while demonstrating how the combination of DR, SPA, and Transformer produces synergistic improvements that exceed their individual contributions. In fault mode analysis, we identify which performance aspects are most affected when specific components are removed.

In summary, Table 5 clearly verifies that the deep reinforcement learning module ensures global policy optimality, the spatiotemporal attention mechanism strengthens dynamic change and key obstacle perception, and the Transformer provides powerful long-term sequence modeling and global dependency awareness. The collaboration among these components enables the proposed method to consistently achieve optimal performance and efficiency across various complex environments, demonstrating excellent scalability and generality, and forming a vital foundation for building high-performance intelligent obstacle avoidance systems.

## 5 Discussion and conclusion

The end-to-end robot intelligent obstacle avoidance method proposed in this paper, based on deep reinforcement learning and a spatiotemporal Transformer architecture, demonstrates excellent performance across multiple simulation environments and when evaluated on real-world sensor data. It consistently outperforms existing mainstream methods in simulation-based evaluations in terms of obstacle avoidance success rate, path efficiency, decision response speed, and model lightweight design. Notably, while our method shows promising results in simulation environments and when tested on real-world sensor data, comprehensive validation in actual physical deployment scenarios remains to be conducted. However, two critical limitations specific to our framework require immediate attention. First, our spatiotemporal attention mechanism exhibits domain sensitivity during sim-to-real transfer, where attention weights learned on simulated obstacle patterns may not effectively transfer to real-world sensor noise and lighting variations. This limitation is particularly pronounced in our Transformer encoder's spatial attention computation (Equations 14–16), where synthetic depth data characteristics differ significantly from real sensor outputs. Second, while our integrated architecture achieves superior performance, the computational overhead of multi-head attention combined with experience replay creates latency bottlenecks that limit real-time deployment on resource-constrained robotic platforms, particularly affecting the 92.37 ms inference time requirement for safe obstacle avoidance.

Based on these specific limitations, we identify two concrete research directions for immediate improvement. First, developing domain-adaptive spatiotemporal attention mechanisms that can automatically adjust attention weights during deployment through meta-learning approaches. This involves creating attention regularization techniques that maintain spatial-temporal modeling capabilities while adapting to real-world sensor characteristics, potentially through adversarial training between simulated and real sensor data. The specific implementation would focus on modifying our attention computation (Equation 16) to include domain-invariant features. Second, architectural optimization for embedded deployment through selective attention pruning and quantization techniques specifically designed for our Transformer-DQN integration. This involves developing attention head importance scoring to identify which spatial-temporal attention patterns are most critical for obstacle avoidance, enabling targeted compression without performance degradation. These improvements directly address our framework's deployment challenges while maintaining its core spatiotemporal modeling advantages.

In summary, although our spatio-temporal Transformer-DQN framework has made progress in simulated environments, addressing specific challenges such as domain transfer sensitivity and computational efficiency is crucial for achieving robust real-world deployment. These targeted improvements represent the most impactful next steps in translating our theoretical findings into practical robot navigation systems.

## Data Availability

The original contributions presented in the study are included in the article/supplementary material, further inquiries can be directed to the corresponding author.
